# Advocacy in Sports Cardiology: A Call to Arms∗

**DOI:** 10.1016/j.jacadv.2024.100985

**Published:** 2024-05-07

**Authors:** Elizabeth H. Dineen, Michael Lawrence, Mustafa Husaini, Alfred Danielian, Peter Dean, Lindsay Davis, Kenneth Edmonds, Eugene H. Chung, Jonathan H. Kim, Dermot M. Phelan

**Affiliations:** a Mayo Clinic, Department of Cardiovascular Medicine, Jacksonville, Florida, USA; b American College of Cardiology, Washington, District of Columbia, USA; c Division of Cardiology, Washington University in St. Louis, St. Louis, Missouri, USA; d Division of Sports Cardiology, Las Vegas, Nevada, USA; e Division of Pediatric Cardiology, Department of Pediatrics, University of Virginia, Charlottesville, Virginia, USA; f Lindsays Law, New York, New York, USA; g National Football League, New York, New York, USA; h Massachusetts General Hospital, Harvard Medical School, Boston, Massachusetts, USA; i Emory Clinical Cardiovascular Research Institute, Emory University School of Medicine, Atlanta, Georgia, USA; j Gragg Center for Cardiovascular Performance, Sanger Heart & Vascular Institute, Atrium Health, Charlotte, North Carolina, USA

**Keywords:** athlete, CPR, defibrillator, exercise, health policy, sudden cardiac death

The sudden cardiac arrest (SCA) or death of a young athlete reverberates far beyond the world of sports. The SCA and successful resuscitation of Buffalo Bill’s player, Damar Hamlin, on live national TV commanded international attention. It ignited demands to understand how such events happen and how they can be prevented. A frequently posed question was: *What can I do to help?* While preparticipation screening is a tool to mitigate risk, not all SCA cases can be prevented. Thus, secondary prevention with the rapid and appropriate response to an acute emergency must also be prioritized by all in the care of competitive athletes.

Early bystander cardiopulmonary resuscitation (CPR) and the use of an automated external defibrillator (AED) save lives, and success is directly related to the speed of response. Although the efforts of various organizations and local community initiatives to offer AEDs and provide CPR education and training are commendable, more substantial improvements in outcomes will likely require state and national legislation. For example, a significant improvement in the rates of bystander CPR has been demonstrated in those states that have implemented state laws mandating CPR education in high schools.^1^ The American College of Cardiology (ACC), the American Heart Association (AHA), and many other medical societies have taken a proactive approach in addressing these advocacy issues and, partnering with the National Football League (NFL) through the Smart Heart Sports Coalition, are working state-by-state to implement legislation. This is one of several advocacy efforts that we will highlight, along with other ways to engage in communities and make a difference.

The NFL-led Smart Heart Sports Coalition was founded in March 2023 in response to Damar Hamlin’s resuscitated SCA and advocates for all 50 states to adopt evidence-based policies that will prevent fatal outcomes from exercise-related SCA among high school students through 3 fundamental policies: 1) access to an AED (at or within 1-3 minutes of the athletic venue); 2) an emergency action plan (EAP) enacted and rehearsed in accordance with national standards and updated annually; and 3) CPR and AED training for coaches (Smart Heart Sports Coalition). While the components of this initiative are well-established for saving lives, the national implementation has been challenging given the necessary resources required (Table 1). Prior to the rollout of the Smart Heart Sports Coalition, only 7 states had existing policies addressing all 3 of the Coalition components. While in action for only a few months, there has been forward momentum driven by the Coalition with state-level policy (eg, Pennsylvania S.B. 375 and Ohio H.B. 47 are pending in the state legislature). The ACC has participated in Coalition events in Columbus, Ohio, and Harrisburg, Pennsylvania, to raise awareness and advocate for both bills, which has led to bipartisan sponsorship. The Coalition provides a framework for states to unify their advocacy efforts, including allocating NFL Foundation grant funds to support nationwide access to AEDs and CPR education, and while efforts are nationwide, the Coalition is targeting 30 states during the 2023 to 2024 state legislative sessions (AZ, CA, CO, DE, FL, IL, IN, IA, LA, ME, MD, MI, MN, MO, MT, NE, NV, NY, OH, OK, PA, SC, SD, TN, VA, WI).

**Table 1:** Smart Hearts Coalition Legislation by State

	Emergency Action Plan	AED Access	CPR and AED Training for Coaches	CPR Training for High School^2^
Alabama	-	√	√	√
Alaska	-	-	-	-
Arizona	-	-	-	√
Arkansas	√	√	√	√
California	√	√	√	√
Colorado	-	-	√	-
Connecticut	√	√	√	√
Delaware	-	√	-	√
Florida	-	√	-	√
Georgia	√	√	√	√
Hawaii	-	√	-	√
Idaho	-	√	-	√
Illinois	-	√	√	√
Indiana	√	√	√	√
Iowa	-	-	-	√
Kansas	√	-	√	√
Kentucky	√	√	√	√
Louisiana	√	√	√	√
Maine	√	√	-	√
Maryland	-	√	√	√
Massachusetts	√	√	√	-
Michigan	-	-	-	√
Minnesota	-	-	-	√
Mississippi	√	-	-	√
Missouri	-	√	-	√
Montana	-	-	-	√
Nebraska	-	-	-	-
Nevada	-	√	-	√
New Hampshire	√	-	√	-
New Jersey	√	√	√	√
New Mexico	√	-	-	√
New York	√	√	-	√
North Carolina	√	√	√	√
North Dakota	-	√	-	√
Ohio	-	-	-	√
Oklahoma	-	√	-	√
Oregon	-	√	-	√
Pennsylvania	-	-	-	√
Rhode Island	-	√	-	√
South Carolina	√	-	√	√
South Dakota	√	-	-	√
Tennessee	-	√	-	√
Texas	√	√	√	√
Utah	-	-	√	√
Vermont	√	-	√	√
Virginia	-	√	-	√
Washington	√	-	-	√
West Virginia	-	√	-	√
Wisconsin	-	√	√	√
Wyoming	-	-	√	-

The “√” symbol is to note that a policy exists for that topic in the state noted. The “-” means nothing exists in that topic for that state.

SCA education is at the heart of any framework for improving outcomes. In 2017, Lindsay’s Law (Ohio Revised Code 3313.5310, 3707.58, and 3707.59) mandated the following in Ohio: 1) SCA education for student-athletes, parents, and coaches; and 2) a requirement for physician evaluation for any concerning personal or family history prior to engaging in competitive sport. Lindsay Davis, the initiative's namesake and co-author of this Viewpoint, led these extensive endeavors to raise awareness about SCA following her personal experience in returning to sports after being diagnosed with hypertrophic cardiomyopathy. Often spurred by tragedies, organizations founded in the memory of fallen student athletes and others, such as Simons Heart and Eric Paredes Save a Life Foundation, have helped channel life-saving education and resources and/or pushed for legislative change. The ACC advocacy team introduced a 50 state issue campaign mandating CPR as a high school graduation requirement for the better part of the past decade,^1^ with policy mandated in all but a few remaining states (CO, MA, NE, NH, WY).^2^ Working to close the gap in those states without legislation remains a priority.

Besides early and effective CPR, access to and appropriate use of AEDs saves lives. The push for mandatory access to AEDs during sporting events is critical; however, it is important to recognize the real-world challenges of such implementation. This will require significant resources to procure, maintain, and distribute these life-saving devices. Perhaps more importantly, it highlights the disparities that exist in resource-poor regions. While affluent areas might readily meet the AED requirement, underserved communities may struggle to allocate the necessary resources for widespread AED deployment. This glaring inequity underscores the importance of comprehensive, equitable, and sustainable strategies for implementing AED access mandates across the United States. The 2023 Access to AEDs Act (H.R.2730) requires the Department of Health and Human Services to award grants to promote student AED access at elementary and secondary schools. As of 2016, 17 out of 50 U.S. states required AEDs in some of their schools, with mixed legislation in private versus public and elementary versus high schools.^3^

Developing a comprehensive EAP is an essential component of ensuring an appropriate response for both athletes on the playing field and general students in the classroom.^4^ It involves identifying key personnel and their roles, ensuring access to life-saving equipment like AEDs, and coordinating a multidisciplinary team for a well-rehearsed response. EAPs are vital for quick and effective responses during catastrophic situations in sports. Implementation of a well-rehearsed EAP was demonstrated on live TV during the aforementioned cardiac arrest of Damar Hamlin; this component is a key part of the Smart Heart Sports Coalition. The AHA is leading an initiative to work with policymakers on including Cardiac Emergency Response Plan language in state policies for EAPs, as many states have broad plans in place that include natural disasters and medical emergencies not specific to SCA. ACC has also placed emphasis on advocacy work at the state level specific to EAP implementation, particularly the need for EAP practice drills in schools (eg, Georgia H.B. 874 is pending in the state legislature as of January 2024). Indeed, beyond the core initial tenets of the Smart Hearts Sports Coalition, a legislative focus must also ensure that an EAP is executed well, without hesitation and additional unneeded chaos. This includes establishing an AED maintenance plan for all schools.

SCA/D education and awareness, CPR training, AED access, and EAP development represent just a few avenues where advocacy efforts can impact the field of sports cardiology and improve the cardiac health of all athletes under our watch. Ensuring all practitioners who are charged in the cardiac care of athletes develop familiarity in this arena is critical, and there are numerous avenues in which one can get involved (Figure). First, one must begin to understand and address health care policies and regulations at the local, state, and national levels. Where are the gaps that exist? Second, it is imperative to stay informed of legislative issues affecting sports cardiology. Which states are enacting new policies, and how are these policies addressed in your state? Third, ensure you are a member of the ACC’s sports and exercise cardiology section and begin to work with the ACC's advocacy team, which seeks to collaborate with clinicians, policymakers, and other stakeholders to shape discussions and formulate supportive legislation for the benefit of patients and the cardiology community. The ACC Advocacy team includes Congressional Affairs, State Government Affairs, Regulatory Affairs, and Payer and Care Delivery components. Covering all 50 states with regional coverage plans, the ACC's State Government Affairs Team collaborates closely with state ACC chapters and policymakers. Fourth, engage with your respective state ACC chapter and the individual state chapter advocacy team, as they play an instrumental role in the Smart Hearts Sports Coalition’s state-level advocacy efforts. Sports cardiologists can invest time in helping to address gaps in state legislation related to athlete health, which can lead to meaningful new laws when partnering with colleagues and policymakers. Fifth, many cardiology organizations have legislative days where members can meet lawmakers and engage in the advocacy process. At ACC, the annual Legislative Meeting in Washington, DC, occurs in the fall and offers a chance to attend unique educational sessions addressing how to work with lawmakers and attend meetings with state representatives and senators. Other organizations often seek volunteers to help organize events, grassroot campaigns, and outreach efforts. Finally, it is important to contact and develop a relationship with policymakers and state legislators. These individuals can be reached through emails, phone calls, letters, and social media. Building relationships with elected officials can lead to opportunities in the future for ongoing cardiology advocacy work.

The prevention of SCA among young athletes requires a multifaceted approach. Legislative initiatives, such as the NFL-led Smart Heart Sports Coalition and the 2023 Access to AEDs Act, play a crucial role in improving outcomes by advocating for widespread CPR education, AED access, and EAP implementation. Collaboration between health care professionals, policymakers, and advocacy organizations, as demonstrated by the ACC, AHA, and other medical societies, is essential for addressing gaps in legislation, promoting athlete health, and ultimately saving lives.

## Funding support and author disclosures

The authors have reported that they have no relationships relevant to the contents of this paper to disclose.
